# Gall-forming aphids are protected (and benefit) from defoliating caterpillars: the role of plant-mediated mechanisms

**DOI:** 10.1186/s12862-021-01861-2

**Published:** 2021-06-18

**Authors:** Lilach Kurzfeld-Zexer, Moshe Inbar

**Affiliations:** Department of Evolutionary & Environmental Biology, University of Haifa, 3498838 Haifa, Israel

**Keywords:** Compensatory leaf growth, Facilitation, Interspecific interactions, *Pistacia*, Processionary moth

## Abstract

**Background:**

Interspecific interactions among insect herbivores are common and important. Because they are surrounded by plant tissue (endophagy), the interactions between gall-formers and other herbivores are primarily plant-mediated. Gall-forming insects manipulate their host to gain a better nutrient supply, as well as physical and chemical protection form natural enemies and abiotic factors. Although often recognized, the protective role of the galls has rarely been tested.

**Results:**

Using an experimental approach, we found that the aphid, *Smynthurodes betae*, that forms galls on *Pistacia atlantica* leaves, is fully protected from destruction by the folivorous processionary moth, *Thaumetopoea solitaria*. The moth can skeletonize entire leaves on the tree except for a narrow margin around the galls that remains intact (“trimmed galls”). The fitness of the aphids in trimmed galls is unharmed. Feeding trials revealed that the galls are unpalatable to the moth and reduce its growth. Surprisingly, *S. betae* benefits from the moth. The compensatory secondary leaf flush following moth defoliation provides new, young leaves suitable for further gall induction that increase overall gall density and reproduction of the aphid.

**Conclusions:**

We provide experimental support for the gall defense hypothesis. The aphids in the galls are protracted by plant-mediated mechanisms that shape the interactions between insect herbivores which feed simultaneously on the same host. The moth increase gall demsity on re-growing defoliated shoots.

## Background

Plants serve as hosts for communities of insect herbivores and their natural enemies. Insect herbivores that share the same host plant may affect each other directly (e.g. by competition) or indirectly through the changes they induce in the plant’s traits; herbivores induce modifications in plant secondary and primary metabolism, morphology, and phenology [1]. These modifications alter the quantity and quality of resources available to other herbivores that feed simultaneously or sequentially on the same plant. Plant-mediated interactions among herbivores are common and important, as they can operate between spatially and temporally separated species [1–5]. Such interactions can be negative or positive (facilitation via induced plant susceptibility), and they tend to be asymmetric [6, 7]. Induced plant response to herbivory can also affect upper trophic levels, altering the density and composition of predators and parasitoids [8].

Gall-forming insects manipulate the development, anatomy, physiology and chemistry of their host for their own benefit. Galls are usually formed on undifferentiated young plant organs. Within the galls, the developing insects may gain a better nutrient supply and protection from abiotic conditions and natural enemies [9–11]. The galling habit provides a convenient arena to examine plant-mediated interactions between insect herbivores and their enemies. Being surrounded by plant tissue (endophagy), gall-forming insects rely primarily on indirect, plant-mediated mechanisms to deal with competitors, predators, parasitoids and pathogens. Physical and chemical gall traits such as thickness, toughness, hair cover, extrafloral nectaries and secondary metabolites may play an important role in reducing enemy attack [12–17]. There is no doubt the galls provide better nourishment for the insects, but their defensive properties have rarely been tested experimentally [16].

Most studies consider morphological and chemical gall traits to be defense mechanisms against natural enemies [10, 11, 17]. Nevertheless, sessile and long-lasting gall formers engage in intra- and interspecific interactions and competition with other insect (and mammalian) herbivores that feed simultaneously or sequentially on the same plant [18–20]. Gall formers may affect other herbivores in various ways. They may alter sink-source allocation and change the defensive chemical properties and nutritional quality of the shared plants. On the other hand, the performance of gall formers can be affected by induced changes in plant quality following feeding by the free-living herbivore. Gall formers may face the risk of actual consumption or damage by folivorous insects [21]. Another guild of natural enemies of gall-forming insect are cecidophagous species i.e., herbivorous insects that feed on gall tissue [22].

**Fig. 1:**
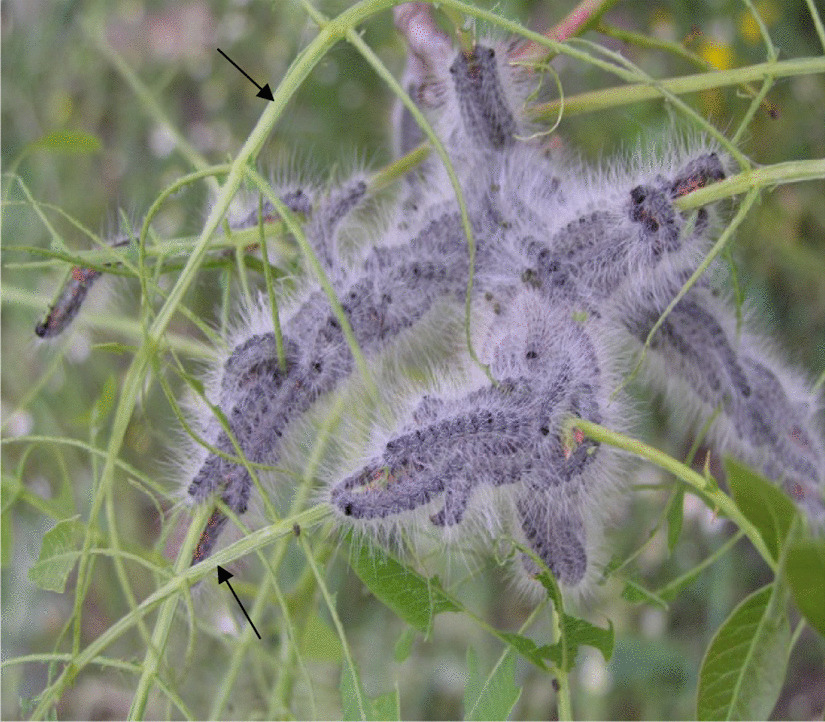
Caterpillars (3^rd^–4^th^ instars) of the processionary moth, *Thaumetopoea solitaria*, feeding in a dense aggregation on *Pistacia atlantica* leaves. Arrows indicate skeletonized leaves

**Fig. 2:**
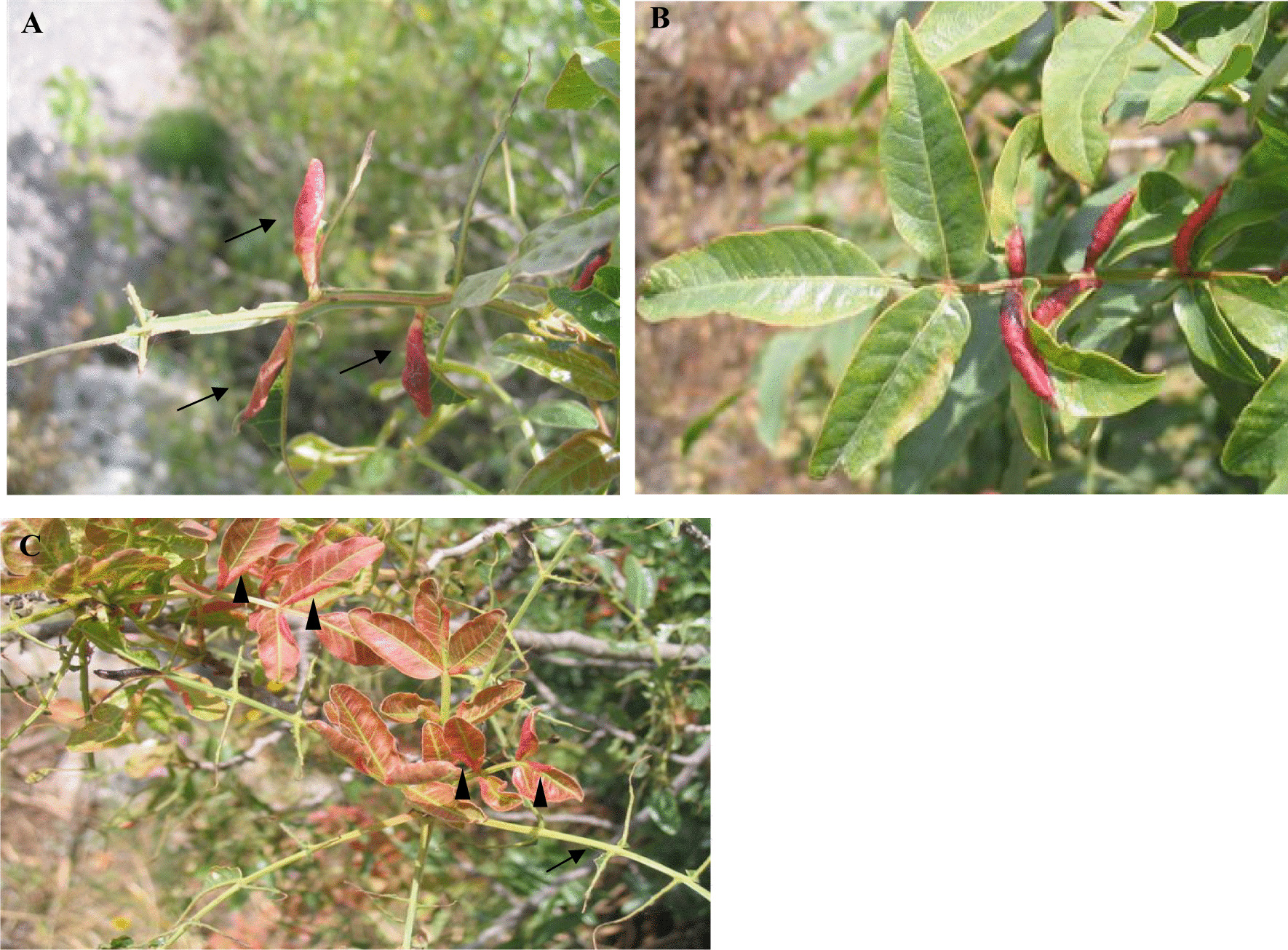
Trimmed (**A**), intact (**B**) and secondary leaf-flush (**C**) F2 galls of *Smynthurodes betae*. The trimmed galls are located on the skeletonized older leaves (arrows) of *Pistacia atlantica* shoots that were defoliated by *Thaumetopoea solitaria*. The leaflets around the galls were consumed by the moths while the galls remain undamaged. The secondary leaf-flush galls (**C**, arrowheads) are located on younger leaves of the same shoots that regrew after moth defoliation. Note the reddish color of the secondary-flush leaves, attributed to their young age

In the spring, aphid species of the tribe Fordini induce galls on young leaves of wild pistachio (*Pistacia* spp.) [23]. At same time, the leaves of *Pistacia* may be completely defoliated by caterpillars of the processionary moth, *Thaumetopoea solitaria* Freyer (Lepidoptera: Thaumetopoeidae) (Fig. 1). Remarkably, the caterpillars that consume and skeletonize entire leaves on the shoots avoid touching the galls, which remain undamaged, hereafter, ‘trimmed galls’ (Fig. 2, see also [21]). Defoliated trees rapidly respond by compensatory growth with a secondary leaf flush ([24]; personal observations). While the moths pupate underground within a few weeks, the aphids continue to develop and reproduce in the trimmed galls for several additional months until the fall.

The main aim of this study was to uncover the mechanisms that affect the density, survival and performance of the common gall-forming aphid, *Smynthurodes betae* West on defoliated shoots. In this species the fundatrix (F1 generation) induces small pea-shaped galls (F1 galls) on the leaflet mid-vein, and her offspring (F2 generation) induce different galls (F2 galls). We first estimated the frequency of host plant sharing by the aphids and the processionary moth. Subsequently, we tested four major hypotheses: 1. The galls are unpalatable and harmful to the caterpillars. 2. Reproduction of the aphids in trimmed galls will be reduced because the caterpillars consume the leaves which are their source of assimilate. 3. Aphids in the exposed, trimmed galls will suffer from greater attack by natural enemies. 4. The compensatory leaf flush of defoliated shoots may provide new, young leaves that can be utilized by late-emerging aphids to produce galls.

## Results

### The frequencies of tree sharing by the moths and the aphids

**Fig. 3:**
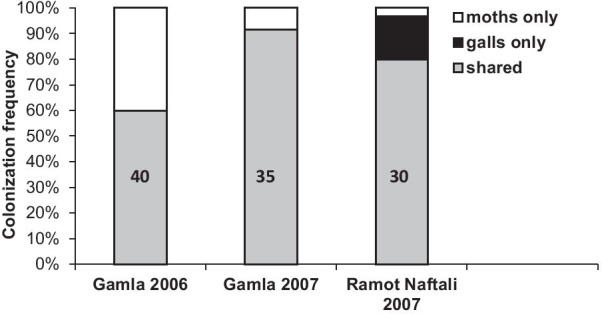
Colonization frequencies of *Pistacia atlantica* trees by *Thaumetopoea solitaria* caterpillars and *Smynthurodes betae* F2 galls, and the frequency of host sharing by both herbivores at the population level at Gamla and Ramot Naftali. The colonization frequencies were calculated out of the total number of trees surveyed in each site and year (numbers in columns)

**Fig. 4:**
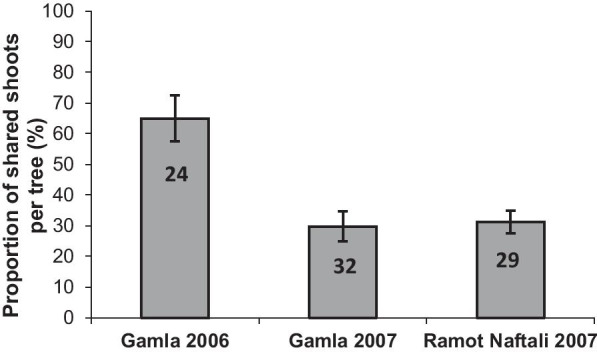
Frequency of host sharing by *Thaumetopoea solitaria* and *Smynthurodes betae* at the tree level at Gamla and Ramot Naftali. The frequency of host sharing is presented as the proportion of shared shoots per tree out of the total number of galled shoots. Only galled trees were included. The data are means (± SEs) per tree. Numbers in columns are sample sizes

Moths and aphids frequently (60–91%) shared the same trees in all sites and years. The frequency of host sharing at the population level in Gamla was higher in 2007 than in 2006 (Fig. 3). At the tree level, the frequency of host sharing was two-fold higher in Gamla in 2006 than in both sites in 2007 (Fig. 4). Likewise, shoot sharing within the same trees in Gamla was significantly higher in 2006 compared to 2007 (Wilcoxon sign test: Z = − 3.179, df = 22, p < 0.001). A similar trend was found when the frequencies of host sharing were evaluated according to the proportion of galls on defoliated shoots out of the total number of F2 galls (Table 1).

**Table 1:** The proportion of *Smynthurodes betae* F2 galls on defoliated shoots (% shared) out of the total number of galls

	Shared	169
Gamla 2006	Total	249
	% Shared	67.9
	Shared	108
Gamla 2007	Total	391
	% Shared	27.6
	Shared	254
Ramot Naftali 2007	Total	876
	% Shared	29.0

### The effects of the moths on the gall-inducing aphids: field experiments

#### The effect of the moths on the distribution and density of F1and F2 galls

**Fig. 5:**
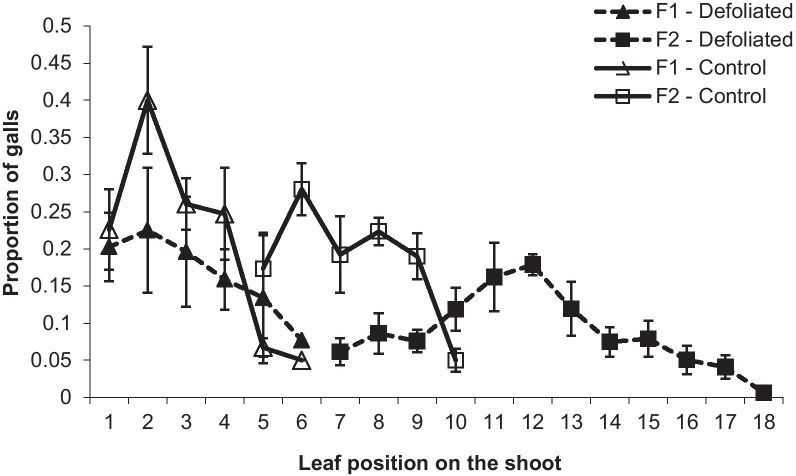
*Smynthurodes betae* F1 and F2 gall distribution on defoliated shoots (including on the secondary leaf flush) compared to control shoots at Gamla. The proportion (mean ± SE) of galls in each leaf position (1 is the oldest, most basal leaf on the shoot and so on) was calculated out of the total number of galls counted on each of the seven trees

**Fig. 6:**
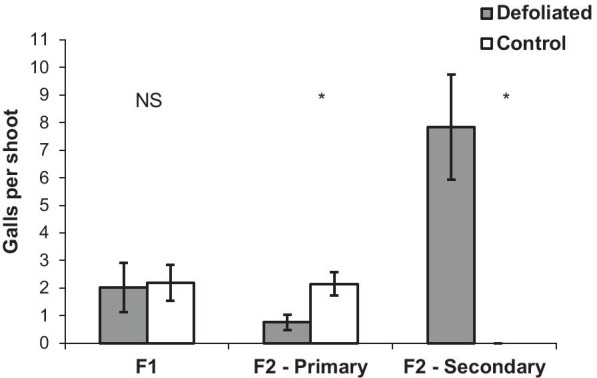
The average of the means of *Smynthurodes betae* F1 and F2 gall densities on seven trees with defoliated and control shoots at Gamla. Data are presented for galls on the primary and secondary leaf flush separately. Differences in each gall type were tested with a paired t-test (*p < 0.01, see text for details)

F1 gall distribution and density per shoot were not affected by the moth (Figs. 5, 6). However, shoots with secondary leaf flush produced up to 18 leaves compared with a maximum of 11 leaves on control shoots. Consequently, additional F2 galls were formed on more distal leaves on secondary leaf-flushed shoots (leaf position 7–18) compared to control shoots (leaf positions 5–10) (Fig. 5). F2 gall density was significantly lower on the primary leaf flush (Paired t_6_ = − 5.9, p < 0.01) but was significantly higher on the secondary leaf flush (Paired t_6_ = 4.1, p < 0.01) on defoliated shoots than on control shoots (Fig. 6). Overall, the mean number of F2 galls per shoot was about four-fold higher on defoliated shoots (Paired t_6_ = 3.8, p < 0.01).

#### The effect of the moths on aphid reproduction

**Fig. 7:**
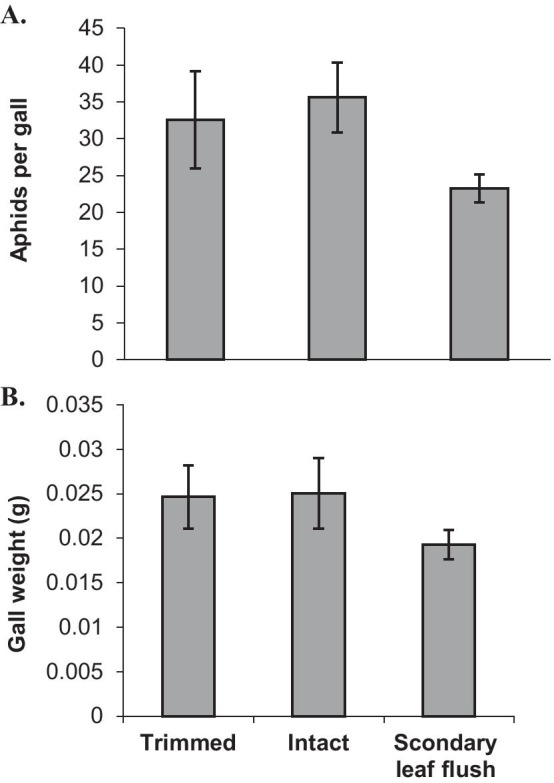
The performance of *Smynthurodes betae* F2 galls: aphid reproduction (**A**) and gall dry weight (**B**) in trimmed and intact galls and in galls from the secondary leaf flush of defoliated shoots in late September, 2006. Data are the average of the means (± SEs) of seven trees. See text for statistical results

Aphid reproduction in control F2 galls was similar (~ 35 aphids/gall) to their reproduction in trees without caterpillars [25]. The mean number of aphids per gall at the peak of reproduction (September) was similar in intact and trimmed galls but clearly lower (by about 35%) in galls induced on the secondary leaf flush (Fig. 7A; repeated measures ANOVA: F_2,12_ = 3.24, P = 0.075). Mean gall weight was similar for the trimmed and intact galls, and lower for the secondary leaf-flush galls (Fig. 7B; Friedman test: χ^2^ = 5.55, P = 0.06). The leaflet area showed a similar trend; it was smaller (about 1.7-fold) in the secondary leaf flush (2.07 ± 0.24 cm^2^) compared to the primary leaf flush of control shoots on the same trees (3.45 ± 0.34 cm^2^) (Paired t_5_ = − 4.4, p < 0.01).

#### The effect of the moths on aphid survival and attack by natural enemies

Predators and parasitoids (some unidentified) may not kill all the aphids in the galls. Hereafter, we refer to survived galls as those that were not parasitized or predated at all or did not die from any other unknown reasons.

In general, gall attack by natural enemies was not affected significantly by *T. solitaria* activity but the source of mortality differed between the three gall types (Table 2). Fungal attack was significantly higher in intact (control) galls. The only identified predatory moth in the galls was * Palumbina guerrini*. Its larvae were less abundant in intact galls than in the trimmed and secondary leaf-flush galls (data not shown). Overall, the rate of attack by predatory moths tended to be higher in the trimmed galls and lower in the secondary leaf-flush galls compared to intact galls (Table 2). The rates of attack by the parasitic wasp * Monoctonia pistaciaecola* and the fly *Leucopis* sp. were similar in all galls. The aphid death due to unknown reasons was lower in the secondary leaf-flush galls (Table 2).

**Table 2:** The percentage of total survival and attack rates of trimmed, intact and secondary leaf-flush F2 galls of *Smynthurodes betae*

Mortality Source	Trimmed (n = 154)	Intact (n = 265)	Secondary leaf-flush (n = 321)	Pearson Chi-Square	df	P
MP	1.95	2.64	5.30	4.51	2	NS
Moths	7.14	5.28	2.80	4.91	2	0.085
*Leucopis* sp*.*	0.65	2.26	0.93	2.64	2	NS
Fungi	7.14	11.70	5.92	6.72	2	< 0.05
Others	12.99	8.30	4.36	11.39	2	< 0.01
Total survival	74.5 ± 6.9	78.2 ± 6.2	84.4 ± 3.5	Repeated measures ANOVA: F_2,12_ = 1.91, NS		

Attack rates were calculated out of the total number of galls examined in all trees (in brackets). Mortality sources: MP = the parasitoid *Monoctonia pistaciaecola*; Moths = *Palumbina guerrini* larvae + moth pupae + holes; *Leucopis* sp*.* = *Leucopis* maggots and pupae; Fungi = unidentified fungal species; Others = empty or dry galls + galls with inquilines

### Feeding experiments

#### Feeding experiments with whole galls

Without an alternative food source, the caterpillars were able to consume whole F2 galls. After two experimental days, most (about 90% of the galls in each jar) were consumed. This indicates that physical properties cannot prevent gall consumption.

#### Feeding experiments with ground galls and leaves

**Fig. 8:**
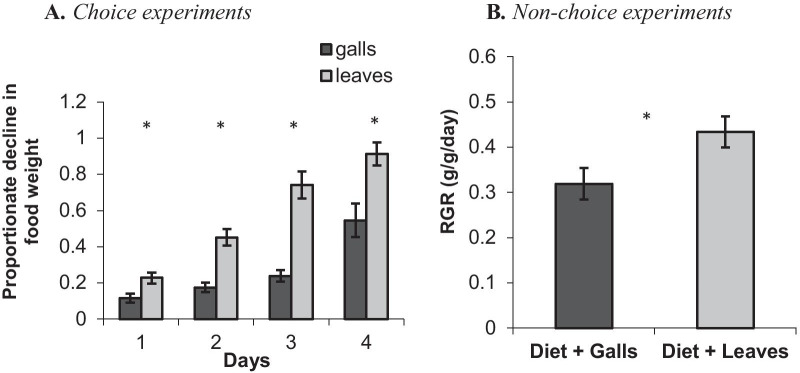
Diet consumption by the caterpillars in the choice experiment (**A**) and caterpillars’ RGR in the non-choice experiment (**B**) with ground *Smynthurodes betae* galls or leaves. Data are means ± SEs of 10 caterpillars per diet. In the choice experiment, differences between galls and leaves in each day were tested with a paired t-test (*p < 0.01). In the non-choice experiment, differences in caterpillars’ RGR after 24 h between the gall and leaf diets were tested with a Student *t*-test (*p < 0.05)

Throughout the choice experiments, the caterpillars preferred leaves over gall tissues. During the first three days, the caterpillars fed mostly on the leaf diet. Only when the leaf diet was fully (or almost fully) consumed (day four), the caterpillars fed more on the gall diet (Fig. 8A). When the caterpillars were forced to feed on galls, their relative growth rate (RGR) was reduced by ~ 25% (Fig. 8B; t_18_ = − 2.26, p < 0.05).

## Discussion

*Pistacia atlantica* is often co-inhabited by the moths and the aphids (Figs. 3, 4; Table 1) at both the whole tree and shoot levels. Occasionally, and especially during outbreaks, intensive moth feeding leaves the trees completely defoliated [24, 26]. Taking into account the intensive defoliation and high frequencies of host sharing, it appears that *T. solitaria* exerts strong selective forces on the survival and performance of Fordini aphids (and their galls). The galls avoid complete destruction by the feeding caterpillars during the spring months, and then maintain their ability to retrieve nutrients from alternative sources long after the caterpillars are gone. We found that the processionary moth and the gall-inducing aphid are engaged in complex, plant-mediated relationships. The gall tissue deters caterpillar feeding, and aphids exploit the production of new leaves after defoliation to induce more galls.

## Why do the moth caterpillars avoid the galls?

The caterpillars feed on the leaves up to a millimeter from the gall edge, which always remains intact. This is true for all gall-forming aphid species on *Pistacia* (MI personal observations, [26, 27]). In nature, once the leaves are scarce, the caterpillars move to different shoots or trees but never feed on the galls. In the lab experiments, without an alternative food source, the caterpillars readily consumed the galls, indicating that their mouthparts can handle the galls’ thickness and toughness. When feeding on ground galls (no physical barrier) the RGR of the caterpillars was significantly reduced (Fig. 8b). We therefore conclude that the chemical properties of the galls play a critical role in aphid (gall) defense. This line of defense deters the caterpillars and reduces their performance.

Insect galls in general accumulate a high level of defensive compounds, especially in their outer layers [28–30]. Compared to *Pistacia* leaves, the galls of Fordini species, including *S. betae*, are loaded with plant-derived defensive metabolites such as tannins, pathogenesis-related proteins, numerous monoterpenes, sesquiterpenes and sticky triterpenes [16, 26, 31–35]. It has been shown that the aphids have the ability to manipulate the metabolic capacity and enzymatic machinery of their *Pistacia* host to produce and accumulate higher levels of monoterpenes for their own benefit [35]. It is likely that not a single compound, but rather the mixture of defensive compounds protects the galls from the caterpillars and other natural enemies [36]. The caterpillars may tolerate, detoxify and even utilize these metabolites that are all present in the leaves, but cannot overcome their higher concentrations in the galls. Our results clearly support the “enemy hypothesis” which emphasizes the protective role of the galls [10, 11, 13, 17].

## Aphid reproduction and survival in trimmed galls

While the moths pupate in the ground by May, the aphids feed and reproduce in the galls for about half a year. Aphid nutrition and reproduction depend on the ability of the gall to act as a physiological sink for assimilates from various plant sources [18, 37–39]. Using ^14^CO_2_-labeling, it was found that galls (of closely related aphid species) that are located on the edges of the leaflets, as F2 galls, normally imported assimilates from the galled leaflet itself [18, 37]. Hence, the main source of assimilates of trimmed galls is gone. The reproductive success of the aphids in the trimmed and intact galls was unharmed (Fig. 7; see also [27]), indicating that these galls are able to import assimilates from more distant alternative sources. The ability of aphids to switch source tissues has been demonstrated in the leaf-margin galls induced by the closely related species, *F. formicaria.* These galls drew assimilates from distal leaflets on the same leaf when their own leaflet was damaged [18]. The caterpillars usually consume the leaves of the entire shoot and even the whole tree (Fig. 1), hence the ability of the small, trimmed F2 galls to maintain their reproduction is quite remarkable. Apparently, during the secondary leaf flush, the trimmed galls can successfully draw or intercept assimilates that are allocated to the new leaves to maintain their vitality.

Contrary to our early hypothesis, the overall attack of F2 galls by natural enemies and their survival were not significantly affected by the caterpillars. The sources of mortality, however, varied between the trimmed, intact and secondary leaf-flush galls (Table 2). These results suggest that the moth-aphid interactions are not affected by the presence of poisonous caterpillar hairs [40, 41], the exposure of trimmed galls or any other plant-mediated chemical modifications.

## Aphids benefit from the plant response to defoliation by the caterpillars

Galls can be induced only on young leaves [42]. Therefore, the synchronization between the insects and their host plant phenology is critically important for gall formation [43, 44]. The supply of suitable leaves for gall induction is a limiting factor for *S. betae* [25, 44, 45]. Fundatrix (F1) gall densities were not affected by the moths because at this point the feeding damage of the first instar caterpillars is rather minimal. F2 (Final) galls are induced on young leaves that emerge successively along the growing shoots. The secondary leaf growth increased the availability of young leaves for the late-emerging F2 aphids. The extended gall-induction window that was released by the moths resulted in four-fold more F2 galls per shoot (Figs. 5, 6).

Gall size and aphid reproduction are positively correlated [42]. Burstein and Wool [44] also found a positive correlation between *S. betae* reproduction and gall size. Generally, larger galls may induce stronger sinks that can support more aphids [37]. Regardless of gall formation, the secondary-flush leaves were smaller than the regular leaves due to limited plant resources. These small leaves supported smaller galls with fewer aphids in agreement with the plant vigor hypothesis that predicts better herbivore performance on large (more vigorous) plant parts [46]. It should be noted that competition between F2 galls over assimilates is weak or absent [44], and therefore could not account for the reduced reproduction in the secondary leaf-flush galls.

Most aphid species produce only one gall, while a few have adopted a two-gall (F1 and F2) strategy [23, 25]. The two-gall strategy reduces the risk of clone extinction due to gall abortion or attack by natural enemies [25, 45, 47]. Here we show an additional adaptive value for this strategy; under natural conditions, *S. betae* have the opportunity to utilize the longer plant growth of the compensatory regrowth after defoliation. Although the reproduction of the aphids in each of these galls was lower, their total reproductive output increased dramatically because most (80%) of the F2 galls were actually induced on the secondary leaf flush (Figs. 5, 6, and 7).

## Conclusions

The interactions between the gall-forming aphids and the caterpillars are primarily plant-mediated. Nutritional selection pressures have undoubtedly shaped the multiple origin of gall formation across insect lineages. This hypothesis has received much research attention, but as pointed out recently by Tooker and Giron [11], there is a lack of experimental evidence on other selective ecological forces. The association between gall defense traits against predators and especially parasitoids is often described, but information on gall defense against other simultaneously feeding herbivores is rather limited [16, 21, 22, 27, 48, 49]. We show that gall defense against co-feeding herbivores is critical to *S. betae* (and related Fordini species), as suggested by Janzen [50]. Defense against insect herbivores that normally feed on intact plant organs should increase the expression and accumulation of defense compounds in the galls by extensive and accurate hijacking of the host metabolism and physiology [9, 51, 52]. We hypothesize that the plant compensatory growth in response to defoliation promotes the evolution of two-gall formation in this group of aphids.

## Methods

### The system

*Pistacia atlantica* Desf. (Anacardiaceae) is a dioecious, deciduous tree with a typical Irano-Turanian distribution, from Central Asia through the Middle East to North Africa. Bud burst begins in March and leaf-fall occurs in October. The common and widely distributed aphid, *S. betae*, induces galls exclusively on leaflets of *P. atlantica*. The complex lifecycle of the aphid includes sexual and parthenogenetic reproduction, and alternation between a primary host (*P. atlantica*) and roots of non-specific secondary hosts on which the aphids do not induce galls. Early in the spring, the fundatrix induces small pea-shaped galls (F1 galls), and her offspring crawl out and induce spindle-shaped, “final” galls (F2 galls) on adjacent young leaflets (Fig. 2). Often the density of F2 galls is limited by the availability of young leaflets. The phloem-feeding aphids reproduce in the galls until the fall and then migrate to secondary hosts [37, 45, 53–55].

The univoltine (one generation/year) processionary moth, *T. solitaria*, is the main folivorous insect of *Pistacia* trees in Israel. Egg hatching is usually synchronous with bud burst in early March. The caterpillars (five instars) feed in dense aggregations on the leaves (Fig. 1) until late May, and then pupate below ground [24, 56]. Occasional population outbreaks of *T. solitaria* might leave the trees completely defoliated ([24], personal observations). The trees response by rapid compensatory re-growth of young leaves.

Fordini galls are attacked by several natural enemies including unidentified pathogenic fungi, insectivorous birds, a parasitic wasp (*M. pistaciaecola*), predatory maggots *Leucopis* sp. (Diptera: Chamamemyiide) and the moth, *Alophia combustella.* Another moth species (*P. guerrini*) is a kleptoparasite, destroying the galls by feeding on their inner tissue [26, 53, 57].

### Frequencies of tree sharing by the moths and the aphids

Forty and 35 trees were surveyed at Gamla Nature Reserve (32° 54′ N, 35° 44′ E) during May 2006 and 2007, respectively (31 were surveyed in both years). Thirty additional trees were surveyed during May 2007 at Ramot Naftali (33° 6′ N, 35° 33′ E). At this time, the caterpillars had already pupated. On each tree, 20–30 shoots were selected randomly. We recorded earlier feeding by caterpillars (“yes” or “no”) according to the presence of skeletonized leaves (Fig. 1), and counted the number of F2 galls on the trees.

The frequency of host sharing was calculated at both population and tree levels. At the population level: [# shared trees/# surveyed trees] per site. Shared trees had at least one shoot with galls and one (not necessarily the same) shoot infested by moths. At the tree level we calculated: (a) [# shared shoots/# galled shoots]; where shared shoots included all shoots that were infested by both galls (at least one gall) and moths, and (b) [# galls on defoliated shoots/total number of galls].

The differences in host-sharing frequencies between the galls and the moths at Gamla and Ramot Naftali were analyzed by independent sample t-tests. The frequencies of host sharing between years at Gamla (on the same tree) were analyzed by paired t-tests or Wilcoxon signed ranks tests, following Kolmogorov–Smirnov tests for normality. The tests were adjusted with a Bonferroni correction, since data from Gamla were tested twice (between sites and years).

### The effects of the moths on the gall-inducing aphids: field experiments

The effects of the moths on the gall density, survival and reproduction of the aphids were examined experimentally on wild populations at Gamla. Galls from defoliated, marked shoots were compared with galls from control (caterpillar-free) shoots that were blocked at their base with Rimifoot, a sticky barrier (RIMI Chemicals Co. Ltd., Israel), that prevents caterpillar access. We examined three gall types: intact (control), trimmed, and those that are induced on the secondary leaf flush (Fig. 2).

#### The effect of the moths on the distribution and density of F1 and F2

Since galls are induced only on juvenile leaves, their distribution along the shoot provides a reliable time scale of their initiation [44]. We recorded the distribution of the galls (F1 and F2) along the re-growing shoots that were defoliated earlier by *T. solitaria*. In early June (no more new galls formed), gall distribution was recorded on seven marked trees that were heavily colonized by *S. betae*. The number of F1 and F2 galls at each leaf position was recorded on 5–7 defoliated, galled shoots, and a similar number of control (blocked) shoots on each tree. The oldest leaf (at the base of the shoot) was marked as leaf #1, and so on. On the defoliated shoots, we distinguished between primary- (often skeletonized) and secondary-flush leaves. The later, younger leaves are softer and reddish in color (Fig. 2).

#### The effect of the moths on aphid reproduction and attack by natural enemies

In late September, at the peak of aphid reproduction in the galls, the trimmed, intact and secondary leaf-flush F2 galls were collected from seven trees. The galls were opened under a dissecting microscope and the number of aphids within was counted. Only galls that were not attacked by natural enemies were examined. The number of aphids per gall is often highly correlated with gall weight [39]. Therefore, gall weight may provide another assessment of aphid success. The empty trimmed, intact and secondary leaf-flush galls were dried at 70 °C for 48 h and then weighed. In addition, we identified and recorded the rate of parasitism and predation on all gall types (trimmed, intact and secondary leaf-flush).

The leaves of the secondary leaf flush appeared smaller. To test this association, additional secondary leaf-flush and control leaves were collected in October from 5 to 10 defoliated and marked control shoots (respectively) in six of the marked trees. The leaflets from each shoot were counted and then pooled for measurement by a digital leaf-area meter (CI-202, CID Inc., Vancouver, WA, USA).

F1 and F2 gall densities on defoliated vs. control shoots within the same tree were compared separately on primary and secondary leaf flush using paired t-tests. The means of all survival and reproduction parameters between trimmed, intact and secondary leaf-flush galls on the same tree were compared using repeated measures ANOVA (“tree” was the repeated factor) or the Friedman test (depending on the Kolmogorov–Smirnov test for normality). Mean leaflet area per shoot on secondary leaf-flush vs. control leaves on the same tree was compared by a paired t-test. The differences in attack rate by natural enemies between trimmed, intact and secondary leaf-flush galls were tested by a Pearson Chi-Square test for independence.

### Moth-feeding experiments

All experiments were conducted with 3rd or 4th instars. Before each feeding experiment, the caterpillars were starved for 24 h.

#### Feeding on whole galls

The ability of the caterpillars to feed on whole galls, i.e., their ability to deal with the gall’s physical traits (size, thickness, toughness) was examined in non-choice feeding trials. In April, shoots carrying young F2 galls were placed individually in 10 jars with water (average 7 galls/shoot). Five caterpillars were placed on each shoot. Since the caterpillar ate leaves only in this set up, we created artificially defoliated shoots with trimmed galls. To force the caterpillar to eat galls, the leaflets around the galls on these shoots were cut with scissors. The number of damaged or consumed galls was recorded during three consecutive days.

#### Feeding on ground galls

These experiments aimed at evaluating the chemical component of gall defense against caterpillars. By using ground galls, we eliminated the physical component of gall defense. Young F2 galls and leaves from the same trees were collected during April and ground with a mortar and pestle using liquid nitrogen. The ground galls and leaves were mixed with an artificial caterpillar food (Instant Soybean-Wheat Germ Insect Diet; “Manduca Premix-Heliothis Premix”; Stonefly Industries, Inc.). The diet dough contained leaves or galls in the same relative amount proportion (40% of total food weight). The doughs were placed in Petri dishes with a single caterpillar (10 replicates for each diet). The caterpillars were weighed before and one day after the experiment, and their relative growth rates (RGR) were calculated. In an additional choice experiments, about 0.5 g dough of each diets (galls and leaves) were placed together in each dish with a single caterpillar. The dough was weighed at the beginning of the experiment and for four days (i.e., 24, 48, 72 and 96 h) afterwards. The amount of food consumed of each diet was calculated daily.

In the non-choice experiment, differences in RGR between caterpillars that were grown on a diet with galls compared to a diet with leaves were tested with a Student’s *t*-test after square-root transformation of the data. In the choice experiment, the proportions of decrease in food weight were compared between gall and leaf diets in the same dishes using a paired t-test after arcsine transformation.

All statistical analyses were conducted with PASW SPSS statistics 17 software. All data were tested for normality prior to statistical analyses using the Kolmogorov–Smirnov test for normality. Only when a normal distribution was found, ANOVA models or t-tests were used. When data did not exhibit a normal distribution, suitable transformations were applied or non-parametric tests were used (as detailed above).

## Data Availability

See link: https://zenodo.org/record/4748265#.YJtl4bUzZaQ.

## Abbreviations

F1 galls: The temporary galls that are induced by the fundatrix (first generation).
F2 galls: The final spindle-shape galls that are induced by the daughters of the fundatrix (second generation)
